# Impact of Remote Monitoring Technologies for Assisting Patients With Gestational Diabetes Mellitus: A Systematic Review

**DOI:** 10.3389/fbioe.2022.819697

**Published:** 2022-03-03

**Authors:** Ayleen Bertini, Bárbara Gárate, Fabián Pardo, Julie Pelicand, Luis Sobrevia, Romina Torres, Steren Chabert, Rodrigo Salas

**Affiliations:** ^1^ Metabolic Diseases Research Laboratory (MDRL), Interdisciplinary Center for Research in Territorial Health of the Aconcagua Valley (CIISTe Aconcagua), Center for Biomedical Research, Universidad de Valparaíso, Valparaíso, Chile; ^2^ Programa de Doctorado en Ciencias e Ingeniería para La Salud, Faculty of Medicine, Universidad de Valparaíso, Valparaíso, Chile; ^3^ School of Biomedical Engineering, Faculty of Engineering, Universidad de Valparaíso, Valparaíso, Chile; ^4^ School of Medicine, Campus San Felipe, Faculty of Medicine, Universidad de Valparaíso, Valparaíso, Chile; ^5^ Cellular and Molecular Physiology Laboratory (CMPL), Department of Obstetrics, Division of Obstetrics and Gynaecology, School of Medicine, Faculty of Medicine, Pontificia Universidad Católica de Chile, Santiago, Chile; ^6^ Department of Physiology, Faculty of Pharmacy, Universidad de Sevilla, Seville, Spain; ^7^ University of Queensland Centre for Clinical Research (UQCCR), Faculty of Medicine and Biomedical Sciences, University of Queensland, Herston, QLD, Australia; ^8^ Medical School (Faculty of Medicine), Sao Paulo State University (UNESP), São Paulo, Brazil; ^9^ Department of Pathology and Medical Biology, University of Groningen, Groningen, Netherlands; ^10^ University Medical Center Groningen (UMCG), Groningen, Netherlands; ^11^ Tecnologico de Monterrey, Eutra, The Institute for Obesity Research, School of Medicine and Health Sciences, Monterrey, Mexico; ^12^ Faculty of Engineering, Universidad Andres Bello, Viña Del Mar, Chile; ^13^ Millennium Institute for Intelligent Healthcare Engineering, Valparaíso, Chile; ^14^ Centro de Investigación y Desarrollo en INGeniería en Salud—CINGS, Universidad de Valparaíso, Valparaíso, Chile

**Keywords:** remote monitoring, telemedicine, gestational diabetes (GDM), technovigilance, mobile applications

## Abstract

**Introduction:** In Chile, 1 in 8 pregnant women of middle socioeconomic level has gestational diabetes mellitus (GDM), and in general, 5–10% of women with GDM develop type 2 diabetes after giving birth. Recently, various technological tools have emerged to assist patients with GDM to meet glycemic goals and facilitate constant glucose monitoring, making these tasks more straightforward and comfortable.

**Objective:** To evaluate the impact of remote monitoring technologies in assisting patients with GDM to achieve glycemic goals, and know the respective advantages and disadvantages when it comes to reducing risk during pregnancy, both for the mother and her child.

**Methods:** A total of 188 articles were obtained with the keywords “gestational diabetes mellitus,” “GDM,” “gestational diabetes,” added to the evaluation levels associated with “glucose level,” “glycemia,” “glycemic index,” “blood sugar,” and the technological proposal to evaluate with “glucometerm” “mobile application,” “mobile applications,” “technological tools,” “telemedicine,” “technovigilance,” “wearable” published during the period 2016–2021, excluding postpartum studies, from three scientific databases: PUBMED, Scopus and Web of Science. These were managed in the Mendeley platform and classified using the PRISMA method.

**Results:** A total of 28 articles were selected after elimination according to inclusion and exclusion criteria. The main measurement was glycemia and 4 medical devices were found (glucometer: conventional, with an infrared port, with Bluetooth, Smart type and continuous glucose monitor), which together with digital technology allow specific functions through 2 identified digital platforms (mobile applications and online systems). In four articles, the postprandial glucose was lower in the Tele-GDM groups than in the control group. Benefits such as improved glycemic control, increased satisfaction and acceptability, maternal confidence, decreased gestational weight gain, knowledge of GDM, and other relevant aspects were observed. There were also positive comments regarding the optimization of the medical team’s time.

**Conclusion:** The present review offers the opportunity to know about the respective advantages and disadvantages of remote monitoring technologies when it comes to reducing risk during pregnancy. GDM centered technology may help to evaluate outcomes and tailor personalized solutions to contribute to women’s health. More studies are needed to know the impact on a healthcare system.

## Introduction

Gestational diabetes mellitus (GDM) is diagnosed when glycemia increases during pregnancy without any previous history (American Diabetes Association, 2020). GDM is currently the most common medical complication of pregnancy and the prevalence of undiagnosed hyperglycemia and even overt diabetes in young women is increasing (McIntyre et al., 2019). In Chile, 1 in 8 pregnant women of middle socioeconomic status has GDM (Garmendia et al., 2020), and 5–10% of women with GDM develop type 2 diabetes after delivery, maintaining a linear growth (Auvinen et al., 2020).

Maternal overweight and obesity (Shin and Sond, 2014), late age at childbearing (Anna et al., 2008), previous history of GDM, family history of type 2 diabetes mellitus, and ethnicity are the main risk factors for GDM (McIntyre et al., 2019). Diagnosis is usually made by an oral glucose tolerance test (OGTT), although in some parts of the world a non-fasting glucose challenge test (GCT) is used to screen for those women who require a full OGTT (McIntyre and Moses, 2020).

In Chile, the diagnosis of diabetes during the first trimester of pregnancy is based on the same criteria used for the general population (World Health Organization, 2006; ADA, 2014). 1) Common symptoms related to diabetes (polydipsia, polyuria, polyphagia and low weight) and a glycemia at any time of the day greater or equal than 200 mg/dl, unrelated to the time elapsed since last meal. 2) Fasting glycemia greater than or equal to 126 mg/dl. Confirm with a second glycemia ≥126 mg/dl, on a different day. 3) Glycemia greater than or equal to 200 mg/dl 2 h after a 75 g glucose load during an OGTT (Ministerio de Salud, 2014).

GDM in the health care system has tripled over a 14-years period, which could be explained by changes in the trend of risk factors during this period (Garmendia et al., 2020), such as the increased prevalence of obesity, along with later fertility which has been associated with higher risk gestation. All this results in an increased burden of care and demand for specialized services (Ministerio de Salud, 2014). For proper management of GDM during pregnancy, it is important to meet the glycemic goals, so patients must achieve constant monitoring, at least once a day, and maybe more, depending on the severity of the glycemia alteration in pregnancy, and they must also comply with dietary treatment and an exercise plan suggested by a specialist (Ministerio de Salud, 2014; McIntyre and Moses, 2020).

The goal of treatment of a woman with GDM is to achieve optimal metabolic control from the time of conception and throughout pregnancy, with fasting and postprandial euglycemia levels, and to screen for and treat any intercurrent pathology (Jovanovic et al., 2005). In Chile, the glycemic target is to maintain fasting glycemia levels between 60 and 90 mg/dl and <140 mg/dl, 1 h postprandial and <120 mg/dl, 2 h postprandial and HbA1c <6% (Ministerio de Salud, 2014).

Keeping a constant and orderly manual record can be complex and cumbersome, considering current lifestyles: women with more than one child often work full or part-time in addition to being homemakers. Thus, to facilitate the constant monitoring of glucose levels or to keep a caloric record of the diet suggested by a specialist, several technological tools have emerged that will make this type of task simpler and more comfortable. This is where the term Telemonitoring or E-Health comes into play, which has made it possible to simplify self-care by empowering the patients themselves to manage their health, while keeping health personnel informed and facilitating access to timely medical care (Lemelin et al., 2020).

The main objective of the review is to evaluate the impact of current technologies and methods of assisting patients with GDM to achieve glycemic goals, and know the respective advantages and disadvantages of these technologies when it comes to reducing risk during pregnancy, both for the mother and her child.

## Methodology

This systematic review was carried out following the guidelines for systematic reviews and meta-analysis (PRISMA) (Urrútia and Bonfill, 2010). To access the literature of interest in a database, in this case, Web of Science, Scopus and Pubmed, it was necessary to identify the search criteria. The following were established as inclusion criteria: Articles related to current technologies for remote monitoring of GDM and parameters and variables related to blood glucose control and treatment compliance monitoring, articles published between 2016 and 2021. As exclusion criteria were established: publications referring to the post-natal/postpartum period, articles related to other types of diabetes, articles related to the diagnosis of GDM, articles related to Pregestational Diabetes Mellitus (PGD), articles from Systematic Reviews and Meta-analyses, articles involving technologies that have not been tested in patients with GDM and articles related to technologies for the use of clinical staff. It should be noted that articles related to GDM treatments and therapies were not excluded from the selection, since a topic of interest in the review is remote monitoring of compliance with these. In addition, there was no exclusion of articles according to the number of study cases, nor will there be exclusion according to the age range of the subjects. After establishing the databases and search criteria, it was important to consider the keywords we used to perform the Boolean expression that gave us the related articles in the databases. To perform the search in the databases, it was necessary to form the optimal search expression that completely covers the topics of interest of the subject to be investigated. Synonyms for gestational diabetes, glycemic levels, and technology or monitoring were chosen among the keywords.

Once the articles that were considered for the review were determined (according to the inclusion/exclusion criteria), the articles were categorized by the type of technology used for remote monitoring of GDM, monitoring parameters, accuracy according to the studies, and by the advantages and disadvantages of the different methods, to estimate which ones might be more practical for patients with GDM.

## Results

### Result Selection

**FIGURE 1:**
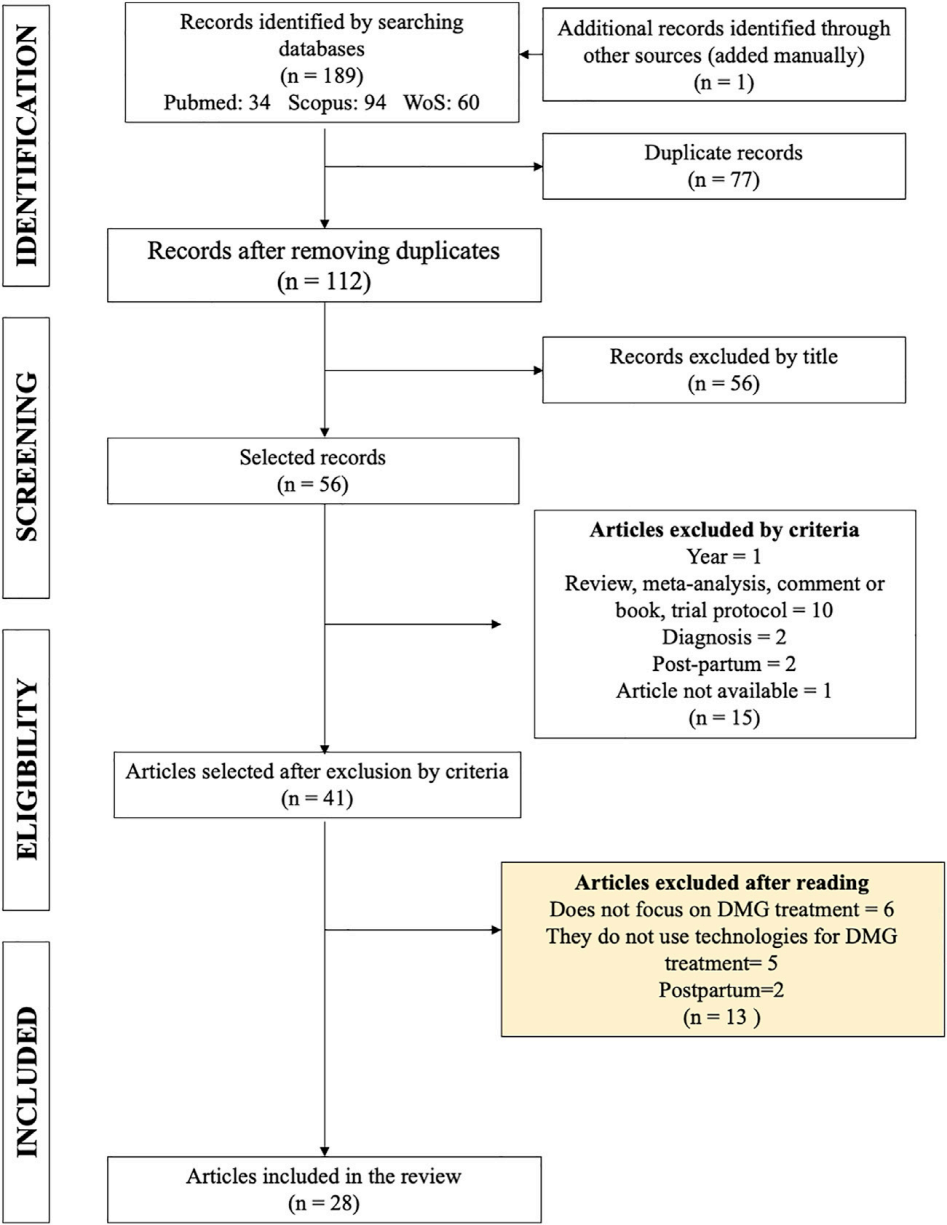
PRISMA diagram for the articles selection process.

The search in the aforementioned databases resulted in a total of 188 articles, and one article was added manually as it met the inclusion criteria (Figure 1), of which 77 were eliminated as duplicates, leaving 112 publications for analysis. After eliminating duplicate entries, the titles were read. From this reading, it was deemed necessary to exclude a further 56 articles with titles too far from the topic of interest. Among the reasons for exclusion of articles by title were: meta-analysis, not using technology, focused on prediction or diagnosis of GDM, studies on mice, not focused on GDM or including the word “postpartum.”

The articles (abstracts) were then analyzed using the list of inclusion and exclusion criteria defined in the Methodology section. As a result, 15 articles that did not meet the guidelines were eliminated: 1 for being outside the stipulated time range; 2 for being concerned with the postpartum period; 2 for focusing on the diagnosis of GDM; 10 articles for being systematic reviews, meta-analyses, commentaries, chapters of books or trial protocols. One article was eliminated because it was not available on any platform. At this point, 41 articles were available for further reading.

Finally, the articles were read, and the decision was made to exclude a further 13 articles. Six of these did not focus on GDM, five did not use technologies for the treatment of GDM and two articles focused on postpartum measurements. In the end, 28 articles were selected for the systematic review (Supplementary Table S1).

### Characteristics of the Selected Studies

Based on the results presented above, we proceeded to analyze each article individually to gather as much information as possible (Table 1).

**TABLE 1:** Main characteristics of selected articles.

Approach of study	Temporality	Geographic location of the study group	Year of publication
Quantitative (85.7%)	Retrospective (21.4%)	Asia (32.1%)	2016 (7.2%)
Qualitative (10.7%)	Prospective (75%)	Europe (46.4%)	2017 (14.3%)
Mixed (3.6%)	Mixed (3.6%)	North America (14.3%)	2018 (35.6%)
		Oceania (7.2%)s	2019 (14.3%)
			2020 (14.3%)
			2021 (14.3%)

Most of the studies were quantitative (24 studies) and prospective (9 studies). Regarding the location of the study group of pregnant women, they were mainly concentrated in Europe (13 studies) and Asia (9 studies). It is important to note that there were no studies that focused on pregnant women in Latin America, the Caribbean, or in Africa. As for the year of publication, the largest number of studies that met the inclusion criteria were published in 2018. Only two studies that met the inclusion criteria were published in 2016.

**FIGURE 2:**
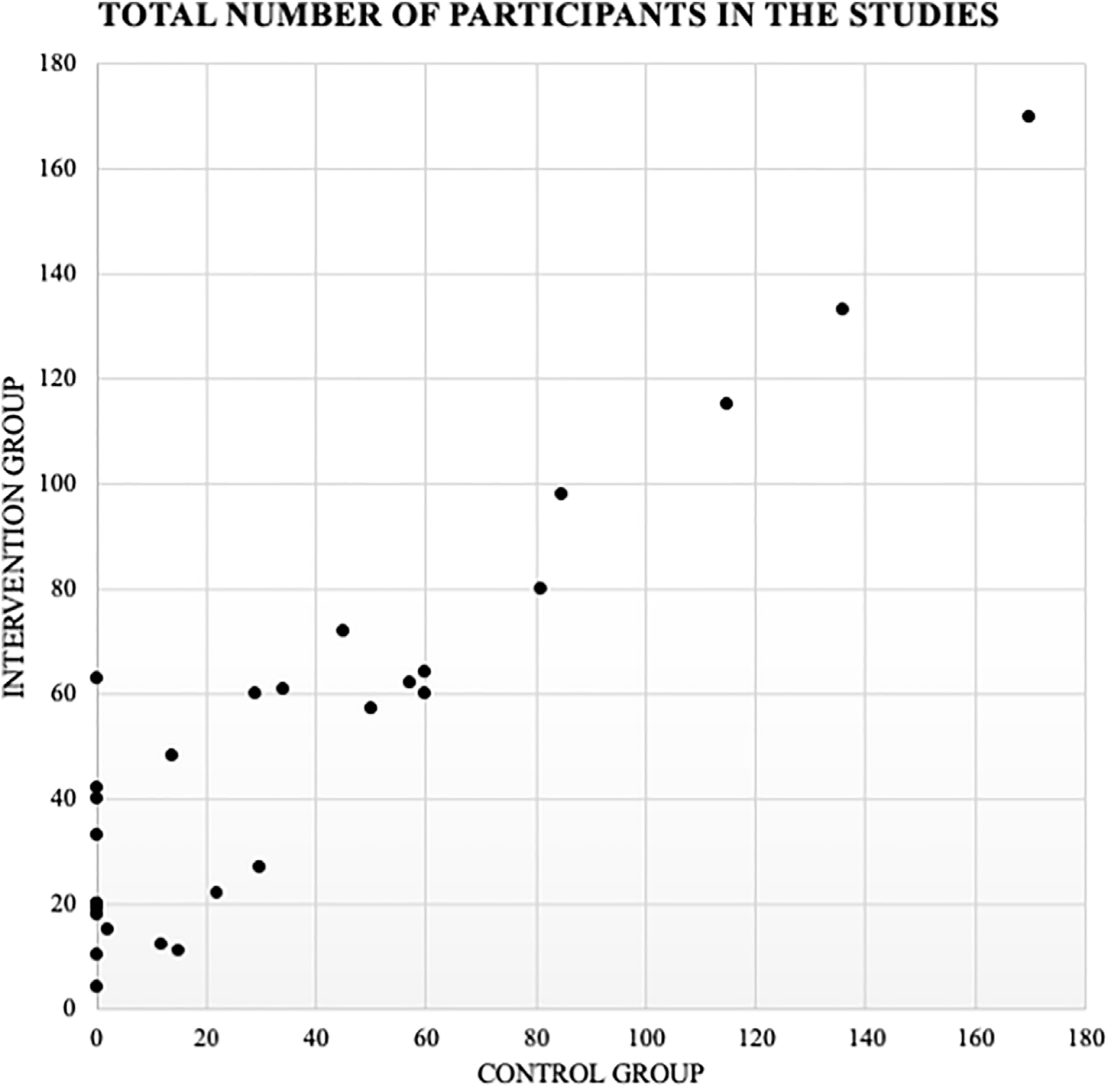
Graphical representation of the distribution of the number of participants. Intervention (with technological intervention for the treatment of GDM) v/s Control (without technological intervention for the treatment of GDM).

Of the total number of articles, 18 of these conducted two study groups or “case controls” where one group did not have the technology intervention for control of GDM and the other did. A total of 9 studies chose only to evaluate the technology tool prospectively, where there is only one study group of intervened pregnant women. One study divides the patients into 3 study groups, separating patients without GDM and with GDM, and separating the latter into those intervened using the technology tool and those not intervened (Figure 2).

### Measurement Parameters

The measurements included in the technology (application) used in the studies include the following characteristics: glycemia (26 articles), bodyweight measurement (7 articles), blood pressure (4 articles), insulin dose (3 articles), number of measurements (2 articles), meals (8 articles), ketonuria (2 articles), physical activity level (7 articles), anxiety and/or depression levels (1 article), medications (1 article), satisfaction levels and quality of life (2 articles). The article that included the technology with the most evaluations included 6 measures: glycemia, weight, blood pressure, ketonuria, physical activity, and satisfaction and quality of life levels (Peleg et al., 2017).

### Medical Devices

Regarding the medical equipment used for glucose monitoring, 11 articles did not specify which one they used or focused on blood results at the beginning and end of the evaluations (Harrison et al., 2017; Johnson and Berry, 2018; Miremberg et al., 2018; Rasekaba et al., 2018; Skar et al., 2018; Triberti et al., 2018; Yang et al., 2018; Kim et al., 2019; Kim et al., 2021; Tian et al., 2021; Varnfield et al., 2021) The most commonly used glucometer was with BlueTooth: This type of glucometer transfers information to the technological device using a Bluetooth integrated into the device. It is necessary that the technological device also have BlueTooth (in this case the most used was a smartphone). It is important to note that, at the time of transferring information, the glucometer should be at a recommended safe distance of 10 m from the technological device. This medical device was used in 6 articles (22.2%) of the total number of articles chosen (Borgen et al., 2017; Peleg et al., 2017; Rigla et al., 2018; Albert et al., 2020; Lemelin et al., 2020; Seo et al., 2020). As for the Smart glucometer, this device measures glycemia just like a conventional glucometer, but must be connected directly by inserting its 3.5 mm jack into the headphone jack of the smartphone. Using the corresponding mobile application, it is possible to perform the measurement and transfer the data to the clinical staff for later review. Three articles (11%) used this medical device (Al-ofi et al., 2019; Guo et al., 2019; Yew et al., 2021). Two articles used a glucometer with an infrared port (Caballero-Ruiz et al., 2016; Caballero-Ruiz et al., 2017), which emits the information to a device reader, which is connected to a computer that will automatically receive the information. This information, after being received by the computer, is uploaded to the platform. It should be noted that the glucometer should be at a distance of 10 cm and the infrared ports facing the front. The continuous glucose monitor requires the insertion of a glucose sensor under the skin, which will receive information that will be continuously transmitted from the patient to the digital platform through the technological device. This medical device was used in two articles (Pustozerov et al., 2018; Pustozerov and Popova, 2018). All studies had moderately invasive methods to achieve glycemic control. Conventional glucometers, smart, Bluetooth and infrared, require blood sampling by the user at certain times depending on each study. The continuous glucose monitor, which goes through the skin, however, does not require the user to be aware of the schedules; she only has to wear it. As for the studies that did not use a glucose monitor, they measured blood glycemia and the other parameters, which implies at least two blood samples per study.

### Technology and Digital Platform

The digital technologies used for GDM monitoring were smartphones, computers and tablets. A total of 14 articles used only a smartphone, 4 articles used a smartphone and computer, 5 articles used a smartphone, computer and tablet, 4 articles used only a computer and one article used a basic phone. The use of these technologies depends on the digital platform used for the telemonitoring of MGD. The articles that used mobile applications relied on smartphones for data collection (Bromuri et al., 2016; Borgen et al., 2017; Harrison et al., 2017; Mackillop et al., 2018; Miremberg et al., 2018; Rigla et al., 2018; Skar et al., 2018; Triberti et al., 2018; Yang et al., 2018; Al-ofi et al., 2019; Guo et al., 2019; Seo et al., 2020; Tian et al., 2021; Varnfield et al., 2021), the articles that used online systems used computers for data collection (Caballero-Ruiz et al., 2016; Caballero-Ruiz et al., 2017; Kim et al., 2019; Wernimont et al., 2020), and the articles that used multiplatform systems used smartphones and computers (Peleg et al., 2017; Albert et al., 2020; Kim et al., 2021; Yew et al., 2021), and four of these also used tablets (Pustozerov et al., 2018; Pustozerov and Popova, 2018; Rasekaba et al., 2018; Alqudah et al., 2019; Lemelin et al., 2020). Only one article did not specify its system used, however, they relied on smartphone data collection (Johnson and Berry, 2018).

### Functions of the Technology for Monitoring GMD

**FIGURE 3:**
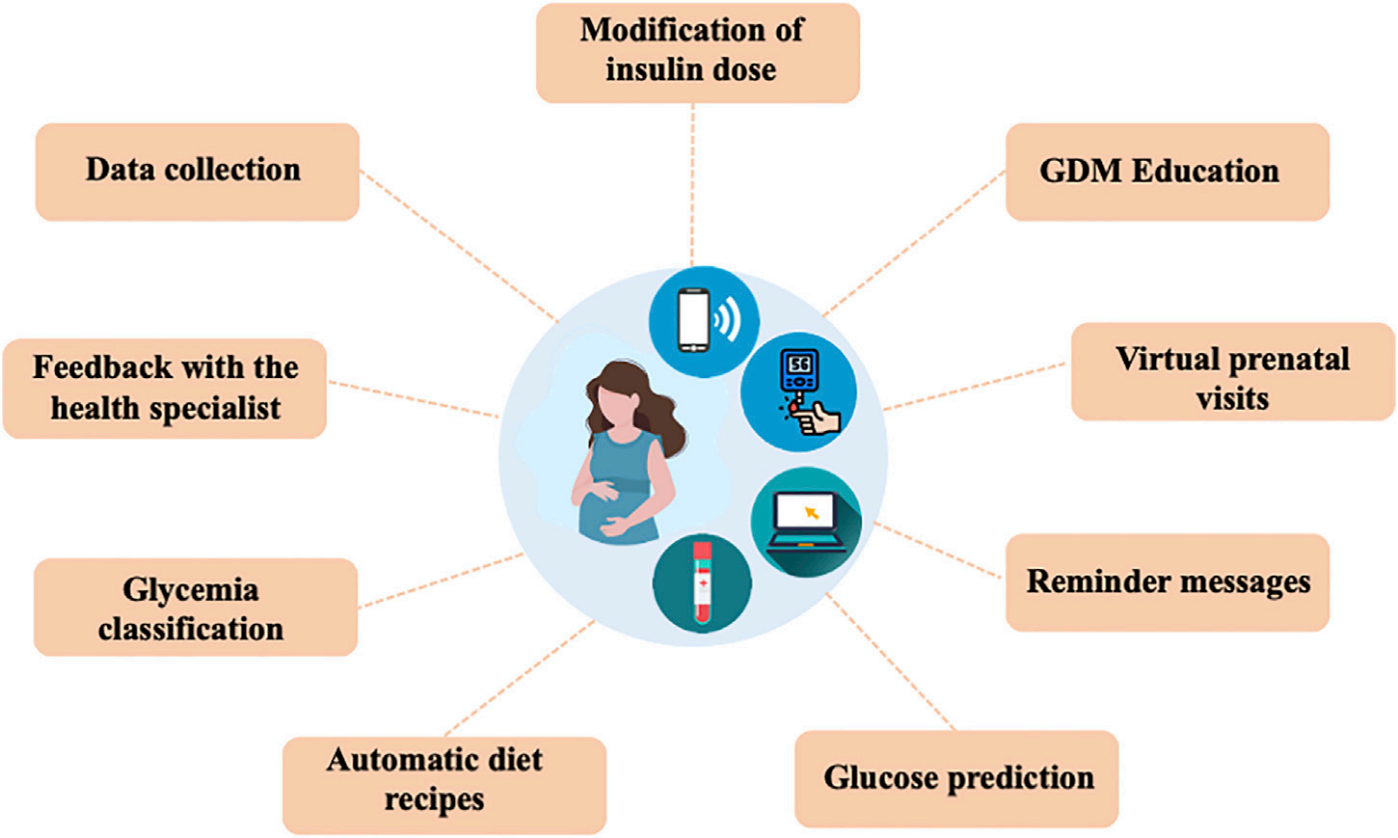
The technological applications were mainly focused on the objective of improving glycemic control and supporting pregnant women in understanding GDM. Nine functions were selected that were common in the applied technologies.

In the selected articles, the technological applications were mainly focused on the objective of improving glycemic control and supporting pregnant women in understanding GDM. Nine functions were selected that were common in the applied technologies: data collection, feedback with the specialist, glycemia classification, automatic feeding recipes, education in GDM, virtual prenatal visits, reminder messages, glycemia prediction and live modification of insulin doses (Figure 3).

#### Data Collection

This function allows the patient to record all values that are requested by the clinical staff. Some are downloaded automatically and others need to be recorded manually. This function is enabled in 81.5% (22 articles) of the total, which is the most repeated (Bromuri et al., 2016; Borgen et al., 2017; Harrison et al., 2017; Peleg et al., 2017; Mackillop et al., 2018; Miremberg et al., 2018; Pustozerov et al., 2018; Pustozerov and Popova, 2018; Rasekaba et al., 2018; Rigla et al., 2018; Triberti et al., 2018; Yang et al., 2018; Al-ofi et al., 2019; Guo et al., 2019; Kim et al., 2019; Albert et al., 2020; Lemelin et al., 2020; Seo et al., 2020; Wernimont et al., 2020; Kim et al., 2021; Varnfield et al., 2021; Yew et al., 2021).

#### Feedback With the Health Specialist

Feedback refers to the communication that exists between the patient and the clinical staff, either through telephone calls, video calls, or text messages. Thirty-seven percent of the articles (10 articles) have this function (Caballero-Ruiz et al., 2017; Peleg et al., 2017; Miremberg et al., 2018; Pustozerov and Popova, 2018; Rasekaba et al., 2018; Triberti et al., 2018; Al-ofi et al., 2019; Albert et al., 2020; Lemelin et al., 2020; Varnfield et al., 2021). In most cases, it goes along with data collection.

#### Glycemia Classification

This type of function allows the patient to automatically associate the appropriate meal and time of measurement (preprandial or postprandial) to each incomplete glucose data downloaded from a glucometer. Five articles (19%) presented this function (Caballero-Ruiz et al., 2016; Borgen et al., 2017; Skar et al., 2018; Seo et al., 2020; Yew et al., 2021).

#### Automatic Diet Recipes

This function delivers automatically personalized diets based on the data recorded by the patients. Like the glycemia classification, this function is present in five articles (19%) (Borgen et al., 2017; Caballero-Ruiz et al., 2017; Skar et al., 2018; Seo et al., 2020; Yew et al., 2021).

#### GDM Education

Through messages or information uploaded to the platform, additional data on diets, exercises, medications, etc. are delivered to treat GDM. Educational information on GDM is presented in 44.4% (12 articles) (Borgen et al., 2017; Johnson and Berry, 2018; Mackillop et al., 2018; Skar et al., 2018; Triberti et al., 2018; Yang et al., 2018; Guo et al., 2019; Albert et al., 2020; Seo et al., 2020; Kim et al., 2021; Tian et al., 2021; Yew et al., 2021).

#### Virtual Prenatal Visits

This intervention makes it possible to alternate the usual clinical visits to the clinic with virtual visits from the patient’s home. This function is enabled in three articles (Harrison et al., 2017; Mackillop et al., 2018; Kim et al., 2019).

#### Reminder Messages

Patients are reminded to perform the corresponding glucose measurements using text messages. This function is found in 7 articles (Borgen et al., 2017; Johnson and Berry, 2018; Skar et al., 2018; Seo et al., 2020; Kim et al., 2021; Tian et al., 2021; Yew et al., 2021).

#### Glucose Prediction

This function allows the prediction of the glucose value without the patient having to measure with the glucometer, all this thanks to the nutritional information recorded by the patient. This function can be found in one article (Pustozerov et al., 2018).

#### Modification of Insulin Dose

The system has automatic responses or responses mediated by health professionals every time the patient needs to modify her insulin doses according to the glycemia measurement. This function is found in two articles (Albert et al., 2020; Yew et al., 2021).

### Glycemic Results

Regarding glycemic results as such, in general, the technological interventions had positive results in the control of GDM. The articles that measured fasting glycemia decreased in the intervention group, in contrast to the control group in four articles (Yang et al., 2018; Guo et al., 2019; Seo et al., 2020; Kim et al., 2021). As for the articles that measured postprandial glycemia, this decreased significantly in four articles that evaluated this measurement (Miremberg et al., 2018; Yang et al., 2018; Al-ofi et al., 2019; Seo et al., 2020). Other studies evaluated glycosylated hemoglobin levels, all of which showed a decrease in this parameter in the experimental group (with technological intervention) versus the non-experimental group (Guo et al., 2019; Kim et al., 2019; Wernimont et al., 2020; Kim et al., 2021) (Table 2).

**TABLE 2:** Results of glycemic control in pregnant women with GDM.

Autor	Control group (n)	Intervetion group (n)	Technology used	Intervention Time	Glycemic control results	Other results
Al-Ofi et al. (2019)	30	27	Smart glucometer + Smartphone + Mobile Application	From week 24 to week 28 the pregnant women began to use the technology (4 weeks). The comparison tests were measured between gestational weeks 38 to 40 (2 weeks)	PPG 2 h is significantly lower in the intervention group (*p* = 0.002)	Most of the pregnant women in the intervention group had adequate gestational weight gain. The weight at the end of pregnancy in the intervention group was significantly lower (*p* = 0.03)
					Fasting glycemia and HbA1c were not significantly different	
Guo, et al. (2019)	60	64	Smart glucometer + Smartphone + Mobile Application	13 weeks	HbA1c before delivery (%) 5.3 ± 0.3 (C) v/s 4.7 ± 0.2 (I) (*p* < 0.001) Off-target fasting glucose measurement (%) 8.3 ± 0.6 (C) v/s 4.6 ± 0.4 (I) (*p* < 0.001) Off-target 2 h post-prandial glucose measurement (%) 14.7 ± 0.8 (C) v/s 7.9 ± 0.7 (I) (*p* < 0.001)	Weight gain after treatment (kg)
						4.8 ± 0.7 v/s 3.2 ± 0.8 (*p* < 0.001)
Yang et al. (2018)	50	57	Smartphone + Mobile Application	From week 24–28 until term (approximately 13 weeks)	FBG (mmol/l) 5.31 ± 1.29 (C) v/s 4.31 ± 0.75 (I) (*p* = 0.000) 1 h PBG	Preterm delivery was significantly less likely in group A than in group B (*p* < 0.05)
					7.75 ± 2.08 (C) v/s 7.71 ± 0.73 (I) (*p* = 0.780) 2 h PBG 6.94 ± 2.47 (C) v/s 5.76 ± 0.67 (I) (*p* = 0.000)	
Miremberg et al. (2018)	60	60	Smartphone + Mobile Application	From week 24–28 until term (approximately 13 weeks)	Mean blood glucose (mg/dl) vs. 112.6 ± 7.4 (C) v/s 105.1 ± 8.6 (I)(*p* < 0.001)Off-target fasting glucose measurement (%) 8.4 ± 0.6 (C) v/s 4.7 ± 0.4 (I) (*p* < 0.001)Off-target 1 h post-prandial glucose measurement (%) 14.3 ± 0.8 (C) v/s 7.7 ± 0.8 (I) (*p* < 0.001) Rate of pregnancies requiring insulin treatment 30.0 (C) v/s 13.3 (I) (*p* = 0.044)	—
Kim et al. (2021)	62	57	Computer/Smartphone + Multiplatform application	12 weeks	Fasting flucose (mg/L) 103 ± 15.6 (C) v/s 92 ± 6.8 (*p* = 0.031) HbA1c (%)5.6 ± 0.3 (C) v/s 5.4 ± 0.3 (I) (*p* = 0.019)	Body weight (kg)
						68.2 ± 17.1 kg (C) v/s 61.5 ± 8.6 (I)
						(*p* = 0.007)
						Body fat (%)
						37.4 ± 5.9 (C) ± 32 ± 5.1 (I)
						(*p*=<0.001)
						Diabetes knowledge
						0.62 ± 0.9 (C) v/s 0.64 ± 0.9 (I)
						(*p* = 0.558)
						Dietary habits
						3.8 ± 0.4 (C) v/s 4 ± 0.3 (I)
						(*p* < 0.001)
						Health Promoting Lifestyle Profile Total Score
						2.64 ± 0.38 (C) v/s 2.82 ± 0.3 (I)
						(*p* < 0.001)
Kim et al. (2019)	22	22	Computer + Web system	12 weeks	HbA1c (%)	Anxiety in the experimental group decreased by 5.1 points but increased by 1.0 points in the control group (*p* = 0.048). Depression increased in both groups
					5.3 ± 0.2 (C) v/s 5.0 ± 0.2 (I)	
					(*p* = 0.001)	
					Glycated albumin (%)	
					11.0 ± 1.4 (C) v/s 10.8 ± 1.2 (I)	
					(*p* = 0.776)	
					Fasting glucose (mg/dl)	
					80.9 ± 8.4 (C) v/s 78.8 ± 8.4 (I)	
					(*p* = 0.075)	
					1 h PBG (mg/dl)	
					117.2 ± 22.2 (C) v/s 129.5 ± 19.9 (I)	
					(*p* = 0.489)	
Seo et al. (2020)	0	4	Glucometer Bluetooth + Smartphone + Mobile application	From week 24–28 until 32—36 weeks (approximately 8 weeks)	Fasting glucose (mg/dl) 104.5 (C) v/s 94.75 (I) 2 h PBG (mg/dl) 223.25 (C) v/s 150.5 (I)	Alteration in the intake of some nutrients:
						1. Reduced consumption of: Calories, proteins, fats, Vitamin A, Vitamin C, Thiamine, Riboflavin, Calcium, animal and vegetable iron
						2. Increased consumption of: Carbohydrates
Wernimont et al. (2020)	45	72	Glucometer smart and standard glucometer (control) + Computer + Web system	From the first prenatal visit until delivery (approximately 34 weeks)	At delivery, women using the cellular glucometer had an average HbA1c of 6.0% compared with an average HbA1c of 6.8% for those women using a standard glucometer. The average decrease in HbA1c from baseline visit to delivery was significantly greater for women using the cellular glucometer (-2.6 ± 1.7%) compared with those using a standard glucometer (-1.4 ± 1.4%)	—

All glycemic outcomes had significant improvements in the intervention groups with the use of GDM monitoring technologies. In addition to glycemic outcomes, there were positive results in terms of lower weight gain during pregnancy (Al-ofi et al., 2019; Guo et al., 2019; Kim et al., 2021), decreased complications at delivery, body fat, dietary habits and Health Promoting Lifestyle Profile Total Score (Kim et al., 2021). Anxiety decreased in the experimental group, but depression increased in both groups (Kim et al., 2019). The consumption of certain nutrients was affected in one of the studies that sought glycemic control through a mobile application (Seo et al., 2020) (Table 2).

### Other Results Associated With the Maternal Perception of the Technologies Used for the Control of GDM

#### User satisfaction and Acceptability

Some of the conclusions of the articles were: Multi-platform system with Bluetooth glucometer monitoring improved user satisfaction (Peleg et al., 2017). In another study, many participants appreciated the ease of access (not having to keep a paper diary), ease of use, and convenience of the mobile application. They liked being able to monitor their blood glycemia values, the application’s ability to connect them quickly, and being able to get answers from their physician (Varnfield et al., 2021). In another study they state that as telemedicine becomes increasingly common in healthcare, user feedback will be essential to tailoring, communicating, and supporting the acceptance and success of these programs (Harrison et al., 2017). In another study, remote glycemia monitoring in women with GDM was shown to be safe. Although glycemic control and maternal and neonatal outcomes were similar, women preferred this model of care (Mackillop et al., 2018). In another study, more than a third of patients gave a cross-platform app with continuous glucose monitoring the maximum score of 10 points for its usefulness in monitoring the disease. At the same time, the convenience of the app received high ratings, with an average value of 8 (Pustozerov and Popova, 2018). Patients who used the system based on a mobile app and Bluetooth glucometer monitoring, according to a survey at the end of the study, reflect a high degree of satisfaction (Rigla et al., 2018). In another study where patients with DM2 and GDM used an app to record glycemic values, they generally adhered satisfactorily to the use of the application (Triberti et al., 2018). In a qualitative study reviewing the perception of mHealth solutions for diabetes in pregnancy, most of these women are willing to self-manage their condition from home and be monitored remotely by a healthcare team (Alqudah et al., 2019).

#### Increased Awareness and Motivation

For many of the women in the study where a mobile app was used, self-monitoring of blood glucose values, including an overview and real-time feedback, was the most important aspect of the app for increasing self-awareness and motivation (Skar et al., 2018). As a conclusion of the study, it is mentioned that there is still much room for improvement in the usability of the multiplatform application associated with continuous glucose monitoring, especially when it comes to perceiving patient motivation (Pustozerov and Popova, 2018).

#### Other Psychological Variables

Patients’ spiritual growth, level of interpersonal relationships and stress management improved significantly (*p* < 0.001) with the use of the cross-platform application-based system (Kim et al., 2021). In another study, anxiety in the experimental group, which used the web system, decreased by 5.1 points and increased by 1.0 points in the control group (*p* = 0.048). Depression increased in both groups (Kim et al., 2019). However, contrary to this result, in another randomized controlled trial, the intervention (multiplatform application) did not increase anxiety or depression (Yew et al., 2021). In another study, patients rated the sense of security provided by the system based on a multiplatform application with Bluetooth glucometer monitoring (Peleg et al., 2017).

### Other Results Associated With the Medical Team Perception of the Technologies Used for the Control of GDM

#### Medical Team Satisfaction and Acceptability

The Bluetooth glucometer-based multiplatform system improved medical team satisfaction (Peleg et al., 2017). Physicians showed high levels of satisfaction with the mobile application (Varnfield et al., 2021).

#### Optimization of Patient Management

Some conclusions from the articles were: The medical team agreed that the multi-platform application and Bluetooth glucometer monitoring facilitated the glycemic management of patients (Peleg et al., 2017). The cross-platform glucometer app can be an excellent tool to avoid unnecessary hospital visits while maintaining better quality medical care and reducing physician workload in the management of GDM (Albert et al., 2020). In another study, all physicians either strongly agreed or agreed that the mobile application improved their efficiency in caring for their patients (Varnfield et al., 2021). In another study, the telemedicine system for GDM intervention did not change health care utilization or clinical outcomes compared to usual care (Rasekaba et al., 2018).

#### Improved Glycemic Control Remotely From the Patient

Some conclusions from the articles were: The use of the multiplatform application and Bluetooth glucometer monitoring resulted in high patient compliance with self-measurement. And physicians agreed that it facilitated patient management (Peleg et al., 2017). In the Smartphone group, there were more glucose measurements, and these measurements were significantly lower compared to the control group (Miremberg et al., 2018). The time needed to achieve optimal glycemic control was significantly shorter for participants in the intervention group using the web-based system than for those using usual care alone. Along with this, women in the intervention group required fewer insulin titrations than controls (Rasekaba et al., 2018). In another study where patients with DM2 and GDM used a mobile app to record glycemic values, there were no significant differences in the recording of glycemia between the two groups (Triberti et al., 2018). With the use of a mobile application, patients in the study showed greater compliance in glucose measurements (Rigla et al., 2018).

#### Time Spent by Clinicians in Patient Assessment and Evaluation

One article showed an 88.6% reduction in face-to-face visits and a 27.4% reduction in time spent by clinicians evaluating patients in the intervention group that used a multiplatform application with Bluetooth glucometer monitoring. The system detected all situations requiring therapeutic adjustment, generating safe recommendations (Albert et al., 2020). The use of a web System reduced personal visits, as well as the time physicians spend evaluating patients, this improves physicians’ efficiency in overcoming their increasing workload (Caballero-Ruiz et al., 2017). It was seen in another study that the *telehomecare* intervention group (THC) had an average of 1.5 versus 3.3 more medical visits than the control group. It increased 10 times more group nursing interventions compared to the control group, promoting greater GDM education. The results of this study show a significant decrease in medical visits and total health care costs for women in the THC group (Lemelin et al., 2020).

### Some Problems or Adverse Study Results

#### Glucose Measurement

Some patients experienced discharge problems with the glucose meter (Albert et al., 2020). In another study, many women experienced technical problems in using the application. Several had problems with the automatic transfer of blood glucose values to the application, and many stopped using the application to record blood glucose values because of this (Skar et al., 2018).

#### Lack of Commitment of Medical Personnel to the Application

The pregnant women stated that the health professionals had little knowledge about the application and that they could not help them when they had problems with the application. Women also reported that their health professionals seemed to have little interest in the application and that they seemed more comfortable looking at blood glucose values on paper, which is standard procedure in the treatment of GDM. Some women stopped using the application to record blood glucose values because their health professionals only looked at their books with the recorded levels and not the application. The lack of support from their health professionals generated some frustration (Skar et al., 2018).

#### Frustration and Misinformation From the Patient

In a qualitative study of system use, some patients admitted that they sometimes “cheated” to get better values and feedback comments. One patient reported waiting 10 min to take her blood sugar to see if the value was lower. In addition, this same app generated some frustration and stress in the users. They stated that it is stressful to think about blood glucose values all the time, and it is frustrating to think if they are out of range. These negative feelings were associated with women who had problems controlling their blood glucose values. Of the women who had to use insulin, none used the application to monitor their blood glucose values, as they saw it as a burden (Skar et al., 2018).

## Discussion

The systematic review conducted provides valuable information to be able to apply telemedicine to pregnant women with GDM. Moreover, it may lay the groundwork for revising technological monitoring of other diseases in pregnancy. One of the strengths of this work was the robust and rigorous search strategy used to find the selected articles since we searched for content in 3 popular repositories that store a large number of current scientific papers, and the methodology explicates in detail what we want to find. This work also allows us to identify which devices and technologies are currently being used and how they are being used (concerning measurements and functions), in addition to describing in detail the main conclusions of the studies; not only for glycemic control and treatment of GDM, but also other characteristics of the use of technological systems, such as user satisfaction, measurements of psychological variables in pregnant women, motivation for using said systems, perceptions of the medical team, optimization of care times, and others. It also includes the possible errors that may be involved in the use of these systems.

Most of the studies were prospective and case-control studies, which reflects good robustness in terms of the quality of the results. Ideally, this type of study should be multicenter to further support the results; however, its application can be difficult because they are systems or studies that require large financial resources.

An important detail to consider is that there are no records in Latin America, so a parallel search was performed and a Brazilian author was found with several publications, mainly on intelligent mobile systems for pregnancy care and prediction, and decision making systems. Both systems presented in the articles found showed high accuracy of 80%, in predicting hypertensive disorders, so they are good predictive methods, but in both articles concluded that more studies were needed [Moreira et al., 2016a; Moreira et al., 2016b (1)]. It is important to encourage this type of study in the population of pregnant women in Latin America because overweight and obesity is a major problem among women of reproductive age in Latin America and the Caribbean, where it is estimated that 70% of women between 20 and 49 years of age are overweight or obese, which are risk factors for GDM. According to the International Diabetes Federation, approximately 12% of live births in Latin America and the Caribbean may be affected by hyperglycemia during pregnancy (Organización Panamericana de la Salud. Hiperglucemia y embarazo en las Américas, 2016; WHO et al., 2019) which is why it is relevant to invite the authors to continue searching for technological strategies for monitoring GDM, based on the results observed in the present review.

It is important to consider that inadequate management of gestational diabetes can lead to various complications, including increased likelihood of a large-for-gestational-age newborn, cesarean section, increased fetal insulin levels, and neonatal fat levels (Coustan et al., 2010). Elevated glycosylated hemoglobin levels were also associated with preeclampsia and preterm delivery (Lowe et al., 2012). On the other hand, a higher maternal BMI, independent of maternal glycemia, is strongly associated with a higher frequency of pregnancy complications, particularly those related to excess fetal growth, adiposity and preeclampsia (Metzger, 2010). Therefore, it is relevant that technologies for the management of GDM should include intake control, weight control and energy and nutrient adequacy to control weight gain in pregnancy.

The categories used in the monitoring of GDM using a technological system coincided precisely with the models of prevention and treatment of GDM: healthy eating and carbohydrate counting (Yuen, 2015; Awuchi et al., 2020), exercise (Mottola, 2008; Bianchi et al., 2017) and lifestyle changes (Moholdt et al., 2020).

In the case of manual transfer of information with standard glucometers, a margin of error may occur at the time of transcribing the value to the digital platform. On the other hand, glucometers with an infrared port must have an additional device (a device reader) for direct transfer to occur. Continuous glucose monitoring (CGM) is becoming increasingly reliable and has demonstrated efficacy in terms of improving glycosylated hemoglobin, reducing hypoglycemia and improving time in the target glucose range. This system that provides immediate feedback to patients and decision support tools for patients and providers have demonstrated superior results compared to intermittent self-monitored blood glucose monitoring (Reddy et al., 2020). More information is needed to know whether the Smart glucometer offers the same reliability as a glucometer with Bluetooth. Just by chance, it is estimated that the most convenient glucometer for pregnant women would be the Bluetooth glucometer since the download is automatic and direct to the technology (as long as this technology has a BlueTooth connection).

There are several factors associated with which platform would be the most convenient. One of them is the need for an internet connection, for example, to access a web system it is necessary to be connected to the internet, while a mobile application may require a connection for a limited time. On the other hand, to be able to download a mobile application, it is necessary to have enough storage space in the digital technology, so it must be a lightweight application. Ideally, the platform should be available in both forms (web page and mobile application), many of the studies reviewed used multiplatform applications, which is the most advantageous concerning the usability of the technology (Delia et al., 2015).

In the selected articles, the technological applications were mainly focused on the objective of improving glycemic control and supporting pregnant women in understanding GDM. These functions are relevant and it is good that they are included in the monitoring of the GDM with the use of technologies. According to the literature, GDM can have serious effects if not adequately treated. An important part of the management of GDM involves patient education on diet, exercise, self-monitoring of blood glucose and self-administration of insulin (Evans and Patry, 2004). It is precisely these functions that the authors of the reviewed articles seek to implement in their systems. It is important to note that none of the selected articles has focused on evaluating the impact of a specific diet on gestational diabetes using these technologies. As future work, it will be of great relevance to analyze various types of diet, such as the effect of a very low-calorie diet through remote monitoring of gestational diabetes.

According to the results found, only 30% of the studies showed glycemic monitoring figures; of these, 100% showed improvements in the measurement parameters: fasting glycemia, postprandial 1 or 2 h, HbA1c, % of measurements outside the recommended range, etc. Even so, it can be concluded that the use of technologies in pregnant women with diabetes is promising; however, studies that continue to support their efficacy in a multicenter, randomized and controlled manner are lacking. It has been previously demonstrated in 11 systematic reviews and 15 meta-analyses, most focused on patients with type 1 diabetes (10 and 6, respectively), reported a reduction in glycosylated hemoglobin (HbA1c) levels from 0.17 to 0.70% after the use of diabetes monitoring systems. Among the control systems is conventional monitoring with traditional glucometer, continuous glucose monitoring, noninvasive glucose monitor, artificial pancreas, insulin pump with sensors and mobile technology or telemedicine (Kamusheva et al., 2021). In this review, we have focused mainly on mobile technology or telemedicine for diabetes monitoring in pregnancy, so the results contribute to this area.

The satisfaction of the patients and the medical team was demonstrated, which provides positive aspects to the use of technology in GDM. What patients highlighted the most was the short time required to use this type of service, in addition to the comfort they provide at the time of monitoring. This empowers patients for their health, increasing their security and confidence concerning their pregnancy condition since they know how they are currently and how to improve their quality of life for the benefit of themselves and their children. On the part of the medical team, what was most valued was the optimization of the time spent on education and monitoring, as well as the greater amount of data associated with glycemic control that they were able to obtain with the applications used.

Some limitations or negative aspects of the use of the technology were concerning glucose measurement, mainly associated with technical problems, though also with the users’ feeling that the clinical staff was not sufficiently committed to the application, which caused them some frustration. Others were associated with the fact that the information using standard glucometers could be modified or altered, or that the pregnant women did not take their glycemia as specified according to the instructions, which could lead to untruthful information at the time of analysis. It was also observed that the use of technology could generate stress in pregnant women due to the excess of information to be processed (both in terms of content and measurement taking).

### Limitations and Future Projections

As for weaknesses of this work, because it details in such a specific way what we want to find, with the inclusion criteria limiting the articles to those published in the last 6 years, there is a probability that texts were left out which could have provided more information regarding remote monitoring of GDM. Also, in the exclusion criteria, the post-natal period is left out, so it cannot be deduced whether the use of technologies decreases maternal or neonatal risks associated with this period in this work.

Regarding the limitations of this review, we recognize that there is no agreement on which is the most appropriate methodology for screening and diagnosis of GDM or standard treatment protocols, so patients may not be precisely comparable among all trials. On the other hand, although values associated with glycemia obtained in patients were found, there was no single or exclusive measurement parameter, which makes it impossible to compare quantitatively the technologies used in the selected articles. There is also no specific information on the digital technology used; only one article specifies the brand and model of the technology, so we do not know what storage space is required for use of the platform, or what operating system it is compatible with if it is a mobile application.

It is important to consider how all the information that can be collected by the digital platforms discussed above can help us. From this arises the definition of Big Data: data sets whose size is beyond the capacity of typical database software to capture, store, manage and analyze (Bahri et al., 2019). During the search of databases, an article emerged about a prototype data integration system from mobile apps and glucometers that aims to standardize the data and have it stored to assist clinicians in the diagnosis and treatment of GDM, but since it is a prototype, there is no information about the results (Pais et al., 2016). It is also necessary to make way for Artificial Intelligence technologies that help to predict glucose, such as the glucose prediction system mentioned in the article by (Pustozerov et al., 2018), as it would allow minimization of invasiveness that can result from the constant use of a glucometer several times a day, and thus improve the quality of life of patients. It would be interesting to optimize resources in medical care by using predictive models for insulin adjustment based on glycemia monitoring and to take it to a clinical context where its use can be validated and prototyped.

## Conclusion

The objective of this systematic review was to determine the impact of current technologies, and recognize types and methods that assist patients with GDM. At the end of this work, it was concluded that monitoring technologies are safe and at no time did they worsen the glycemic status of pregnant women. However, a greater number of randomized controlled multi-center studies and clinical trials are needed, as well as a standardization of the measurements to be established to consider a given measurement “an improvement in glycemic control”. There were benefits such as increased satisfaction and acceptability, maternal confidence, and knowledge of GDM and thus improvements in the quality of the health service delivered. There were also positive comments in terms of optimizing the time of the medical team. GDM centered technology may help to evaluate outcomes and tailor personalize solutions. Further studies are still needed to understand the efficacy and economic impact that could arise from the use of this type of intervention. The present review provides an opportunity to learn about the use of technology in GDM and contribute to women’s health.

## Data Availability

The original contributions presented in the study are included in the article/Supplementary Material, further inquiries can be directed to the corresponding author.
