# Supplementary material for: Impact of Remote Monitoring Technologies for Assisting Patients With Gestational Diabetes Mellitus: A Systematic Review
Source: Front Bioeng Biotechnol. 2022 Mar 3;10:819697. doi: 10.3389/fbioe.2022.819697 (PMC8929763; doi:10.3389/fbioe.2022.819697)
Supplement: Supplementary file 1 [file Presentation1.pptx]

## Slide 1
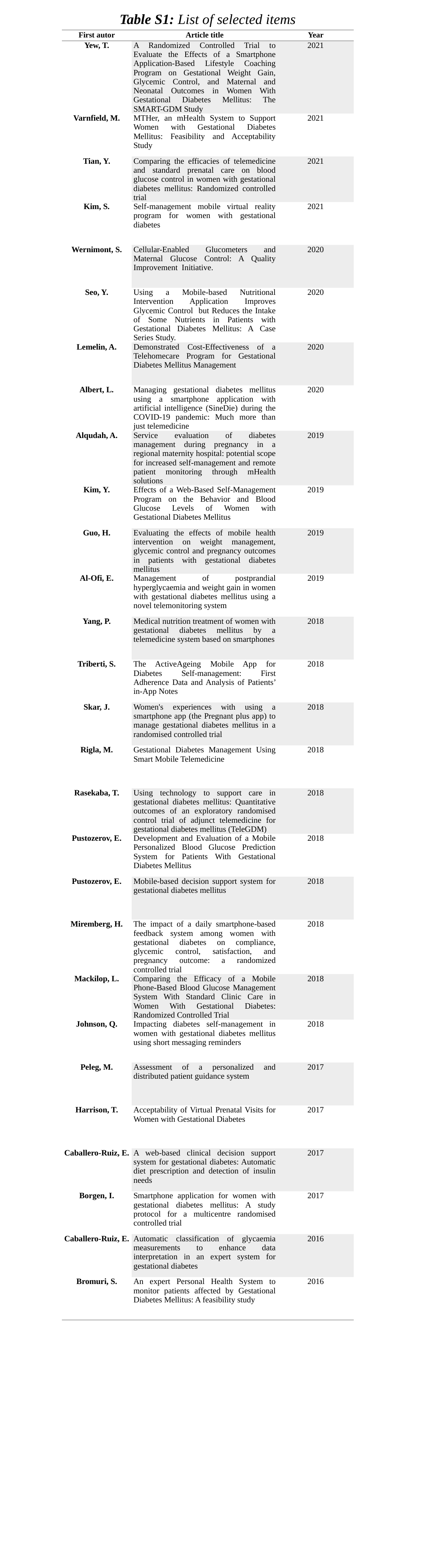

Table S1: List of selected items
| First autor | Article title | Year |
| --- | --- | --- |
| Yew, T. | A Randomized Controlled Trial to Evaluate the Effects of a Smartphone Application-Based Lifestyle Coaching Program on Gestational Weight Gain, Glycemic Control, and Maternal and Neonatal Outcomes in Women With Gestational Diabetes Mellitus: The SMART-GDM Study | 2021 |
| Varnfield, M. | MTHer, an mHealth System to Support Women with Gestational Diabetes Mellitus: Feasibility and Acceptability Study | 2021 |
| Tian, Y. | Comparing the efficacies of telemedicine and standard prenatal care on blood glucose control in women with gestational diabetes mellitus: Randomized controlled trial | 2021 |
| Kim, S. | Self-management mobile virtual reality program for women with gestational diabetes | 2021 |
| Wernimont, S. | Cellular-Enabled Glucometers and Maternal Glucose Control: A Quality Improvement Initiative. | 2020 |
| Seo, Y. | Using a Mobile-based Nutritional Intervention Application Improves Glycemic Control but Reduces the Intake of Some Nutrients in Patients with Gestational Diabetes Mellitus: A Case Series Study. | 2020 |
| Lemelin, A. | Demonstrated Cost-Effectiveness of a Telehomecare Program for Gestational Diabetes Mellitus Management | 2020 |
| Albert, L. | Managing gestational diabetes mellitus using a smartphone application with artificial intelligence (SineDie) during the COVID-19 pandemic: Much more than just telemedicine | 2020 |
| Alqudah, A. | Service evaluation of diabetes management during pregnancy in a regional maternity hospital: potential scope for increased self-management and remote patient monitoring through mHealth solutions | 2019 |
| Kim, Y. | Effects of a Web-Based Self-Management Program on the Behavior and Blood Glucose Levels of Women with Gestational Diabetes Mellitus | 2019 |
| Guo, H. | Evaluating the effects of mobile health intervention on weight management, glycemic control and pregnancy outcomes in patients with gestational diabetes mellitus | 2019 |
| Al-Ofi, E. | Management of postprandial hyperglycaemia and weight gain in women with gestational diabetes mellitus using a novel telemonitoring system | 2019 |
| Yang, P. | Medical nutrition treatment of women with gestational diabetes mellitus by a telemedicine system based on smartphones | 2018 |
| Triberti, S. | The ActiveAgeing Mobile App for Diabetes Self-management: First Adherence Data and Analysis of Patients’ in-App Notes | 2018 |
| Skar, J. | Women's experiences with using a smartphone app (the Pregnant plus app) to manage gestational diabetes mellitus in a randomised controlled trial | 2018 |
| Rigla, M. | Gestational Diabetes Management Using Smart Mobile Telemedicine | 2018 |
| Rasekaba, T. | Using technology to support care in gestational diabetes mellitus: Quantitative outcomes of an exploratory randomised control trial of adjunct telemedicine for gestational diabetes mellitus (TeleGDM) | 2018 |
| Pustozerov, E. | Development and Evaluation of a Mobile Personalized Blood Glucose Prediction System for Patients With Gestational Diabetes Mellitus | 2018 |
| Pustozerov, E. | Mobile-based decision support system for gestational diabetes mellitus | 2018 |
| Miremberg, H. | The impact of a daily smartphone-based feedback system among women with gestational diabetes on compliance, glycemic control, satisfaction, and pregnancy outcome: a randomized controlled trial | 2018 |
| Mackilop, L. | Comparing the Efficacy of a Mobile Phone-Based Blood Glucose Management System With Standard Clinic Care in Women With Gestational Diabetes: Randomized Controlled Trial | 2018 |
| Johnson, Q. | Impacting diabetes self-management in women with gestational diabetes mellitus using short messaging reminders | 2018 |
| Peleg, M. | Assessment of a personalized and distributed patient guidance system | 2017 |
| Harrison, T. | Acceptability of Virtual Prenatal Visits for Women with Gestational Diabetes | 2017 |
| Caballero-Ruiz, E. | A web-based clinical decision support system for gestational diabetes: Automatic diet prescription and detection of insulin needs | 2017 |
| Borgen, I. | Smartphone application for women with gestational diabetes mellitus: A study protocol for a multicentre randomised controlled trial | 2017 |
| Caballero-Ruiz, E. | Automatic classification of glycaemia measurements to enhance data interpretation in an expert system for gestational diabetes | 2016 |
| Bromuri, S. | An expert Personal Health System to monitor patients affected by Gestational Diabetes Mellitus: A feasibility study | 2016 |
